# Supplementary material for: Dissemination of OXA-48- and NDM-1-Producing Enterobacterales Isolates in an Algerian Hospital
Source: Antibiotics (Basel). 2022 May 31;11(6):750. doi: 10.3390/antibiotics11060750 (PMC9220339; doi:10.3390/antibiotics11060750)
Supplement: Supplementary file 1 [file antibiotics-11-00750-s001.zip › antibiotics-1730567-supplementary.pdf]

Supplementary data

Table S1. Antibiotic resistance profiles and phenotypic characteristics of carbapenemase-producing Enterobacterales isolates recovered in out- and in-patients at Annaba University Hospital, Algeria.

Isolate number	Species	Wards	Isolation sites	Antimicrobial resistance[a]	Phenotypic characterization[b]	
					MBL	OXA-48-type
**2490**	*KP*	Outpatient	Urine	AMX, AMC, ERT, IMP	-	+
**859**	*KP*	Haematology	Blood	AMX, AMC, FOX, CTX, CAZ, FEP, ATM, ERT, IMP, MEM, TOB, GEN, OFX CIP	-	+
**2578B**	*KP*	Outpatient	Urine	AMX, AMC, ERT	-	+
**19M3**	*KP*	Intensive Care Unit	Urine	AMX, AMC, FOX, CTX, CAZ, FEP, ERT, MEM, TOB, GEN, AMK, OFX, CIP	+	-
**19V**	*KP*	Intensive Care Unit	Urine	AMX, AMC, FOX, CTX, CAZ, FEP, ATM, ERT, IMP, MEM, TOB, GEN, AMK, OFX, CIP	+	-
**27**	*KP*	Haematology	Blood	AMX, AMC, FOX, CTX, CAZ, FEP, ATM, ERT, IMP, MEM, TOB, GEN, OFX, CIP	-	+
**57**	*KP*	Endocrinology	Pus	AMX, AMC, FOX, CTX, CAZ, FEP, ATM, ERT, IMP, MEM, TOB, GEN, AMK, OFX, CIP	-	+
**51A**	*KP*	Outpatient	Pus	AMX, AMC, FOX, CTX, CAZ, FEP, ATM, ERT, IMP, MEM, TOB, GEN, OFX, CIP	-	+
**51B**	*KP*	Outpatient	Pus	AMX, AMC, CTX, CAZ, FEP, ATM, ERT, IMP, TOB, GEN, OFX, CIP	-	+
**58A**	*KP*	Outpatient	Urine	AMX, AMC, FOX, CTX, CAZ, FEP, ATM, ERT, IMP, MEM, TOB, GEN, OFX, CIP	-	+
**6**	*KP*	Haematology	Blood	AMX, AMC, FOX, CTX, CAZ, FEP, ATM, ERT, IMP, MEM, TOB, GEN, OFX, CIP	-	+
**Z6**	*KP*	Outpatient	Urine	AMX, AMC, FOX, ERT, IMP, MEM, OFX, CIP	-	+
**Z7**	*KP*	Outpatient	Urine	AMX, AMC, FOX, CTX, CAZ, FEP, ATM, ERT, IMP, MEM, TOB, GEN, AMK, OFX, CIP	-	+
**Z9**	*KP*	Outpatient	Urine	AMX, AMC, FOX, CTX, CAZ, FEP, ATM, ERT, IMP, MEM, TOB, GEN, AMK, OFX, CIP	-	+
**Z11**	*KP*	Outpatient	Urine	AMX, AMC, FOX, CTX, CAZ, FEP, ATM, ERT, IMP, MEM, TOB, GEN, AMK, OFX, CIP	-	+
**Z12**	*KP*	Outpatient	Urine	AMX, AMC, FOX, CTX, CAZ, FEP, ATM, ERT, IMP, MEM, TOB, AMK, OFX, CIP	-	+
**Z13**	*KP*	Infectious Diseases	Urine	AMX, AMC, FOX, CTX, CAZ, FEP, ATM, ERT, IMP, MEM, TOB, GEN, AMK, OFX, CIP	-	+
**Z14**	*KP*	Outpatient	Urine	AMX, AMC, FOX, CTX, CAZ, FEP, ATM, ERT, IMP, MEM, TOB, GEN, AMK, OFX, CIP	-	+
**Z15**	*KP*	Haematology	Blood	AMX, AMC, FOX, CTX, CAZ, FEP, ATM, ERT, IMP, MEM, TOB, GEN, OFX, CIP	-	+
**Z16**	*KP*	Outpatient	Urine	AMX, AMC, FOX, CTX, CAZ, FEP, ATM, ERT, IMP, MEM, TOB, GEN, AMK, CIP, OFX	-	+
**Z21**	*KP*	Infectious disease	Urine	AMX, AMC, FOX, CTX, CAZ, FEP, ATM, ERT, IMP, MEM, TOB, GEN, AMK, OFX, CIP	-	+
**Z23**	*KP*	Outpatient	Urine	AMX, AMC, FOX, CTX, CAZ, FEP, ATM, ERT, TOB, GEN, OFX, CIP	-	+
**Z33**	*KP*	Infectious Disease	Urine	AMX, AMC, FOX, FEP, ERT, IMP, MEM, TOB, GEN, OFX, CIP	-	+
**2578A**	*E. cloacae*	Outpatient	Urine	AMX, AMC, FOX, ERT, IMP	-	+
**30**	*E. coli*	Intensive Care Unit	Urine	AMX, AMC, CTX, CAZ, ERT, IMP	-	+
**58B**	*E. coli*	Outpatient	Urine	AMX, AMC, FOX, CTX, CAZ, FEP, ATM, ERT, IMP, TOB, OFX, CIP	-	+

**56**	*E. coli*	Outpatient	Urine	AMX, AMC, FOX, CTX, CAZ, FEP, ATM, ERT, IMP, MEM, TOB, GEN, AMK, OFX, CIP	+	-
**Z18**	*E. coli*	Pediatric	Urine	AMX, AMC, ERT, IMP	-	+
**Z27**	*E. coli*	Intensive Care Unit	Urine	AMX, AMC, FOX, CTX, CAZ, FEP, ATM, ERT, TOB, GEN, OFX, CIP	-	+

* KP, *Klebsiella pneumoniae*; [a]AMX, amoxicillin; AMC, amoxicillin-clavulanic acid; AMK, amikacin; ATM, aztreonam; CAZ, ceftazidime; CIP, ciprofloxacin; CTX, cefotaxime; ERT, ertapenem; FEP, cefepime; FOX, cefoxitin; GEN, gentamicin; IMP, imipenem; MEM, meropenem; OFX, ofloxacin; TOB, tobramycin. [b]KPC/MBL & OXA-48 Confirm (KMOC) Kit.

Table S2. MICs values of carbapenems and colistin for the carbapenemase-producing Enterobacterales.

Isolate number	Species	MICs values (mg/L)			
		**ertapenem**	**imipenem**	**meropenem**	**colistin**
**2490**	*K. pneumoniae*	8	3	0.5	0.125
**859**	*K. pneumoniae*	32	3	8	0.5
**2578B**	*K. pneumoniae*	4	1	0.25	0.25
**19M3**	*K. pneumoniae*	16	3	8	0.25
**19V**	*K. pneumoniae*	32	8	8	0.5
**27**	*K. pneumoniae*	32	16	32	0.5
**57**	*K. pneumoniae*	4	3	3	0.125
**51A**	*K. pneumoniae*	32	8	16	0.25
**51B**	*K. pneumoniae*	1.5	3	1	0.125
**58A**	*K. pneumoniae*	32	4	32	0.5
**6**	*K. pneumoniae*	>32	16	32	1
**Z6**	*K. pneumoniae*	32	4	32	0.5
**Z7**	*K. pneumoniae*	16	8	32	0.5
**Z9**	*K. pneumoniae*	>32	32	16	1
**Z11**	*K. pneumoniae*	32	32	32	0.5
**Z12**	*K. pneumoniae*	8	3	4	0.25
**Z13**	*K. pneumoniae*	>32	4	32	0.25
**Z14**	*K. pneumoniae*	32	4	32	0.5
**Z15**	*K. pneumoniae*	4	4	8	0.125
**Z16**	*K. pneumoniae*	>32	8	32	0.25
**Z21**	*K. pneumoniae*	16	3	16	0.5
**Z23**	*K. pneumoniae*	4	0.125	0.25	0.125
**Z33**	*K. pneumoniae*	32	16	32	0.25
**2578A**	*E. cloacae*	1.5	3	2	0.125
**30**	*E. coli*	2	8	2	0.25
**58B**	*E. coli*	1.5	3	1	0.25
**56**	*E. coli*	32	8	8	0.5
**Z18**	*E. coli*	2	4	1	0.125
**Z27**	*E. coli*	2	1	1.5	0.25

Following the EUCAST Breakpoints (v1.0, 2021), the MICs cut-offs were: ertapenem: >0.5 mg/L, imipenem and meropenem: > 2 mg/L, and colistin: > 2 mg/L
